# Defects, Lithium Mobility and Tetravalent Dopants in the Li_3_NbO_4_ Cathode Material

**DOI:** 10.1038/s41598-018-37466-x

**Published:** 2019-02-18

**Authors:** Navaratnarajah Kuganathan, Apostolos Kordatos, Nikolaos Kelaidis, Alexander Chroneos

**Affiliations:** 1 Department of Materials, Imperial College London, London, SW7 2AZ United Kingdom; 2 Faculty of Engineering, Environment and Computing, Coventry University, Priory Street, Coventry, CV1 5FB United Kingdom

## Abstract

The defect processes of oxides such as self-diffusion impact their performance in electrochemical devices such as batteries and solid oxide fuel cells. The performance of lithium ion batteries can be improved by increasing the Li-ion diffusion. In that respect Li_3_NbO_4_ is identified as a positive electrode material for rechargeable lithium ion batteries. Here, we employ static atomistic scale simulations to examine the defect properties, doping behaviour and lithium ion migration paths in Li_3_NbO_4_. The present calculations show a correct reproduction of experimentally observed crystal structure of Li_3_NbO_4_. The Li-Nb anti-site defect is found to be the dominant intrinsic defect process suggesting that a small concentration of Li on Nb sites and Nb on Li sites is present. Vacancy assisted long range lithium diffusion paths were examined and our calculations reveal that the lowest activation energy (1.13 eV) migration path is two dimensional forming a zig-zag shape. Subvalent doping by Ge on the Nb site is thermodynamically favourable process and a potential strategy to incorporate extra Li in the form of Li interstitial in Li_3_NbO_4_. The results presented herein can motivate further experimental work for the development of Li_3_NbO_4_ based batteries.

## Introduction

Considerable attention has been devoted to the development of novel high capacity cathode materials for rechargeable lithium ion batteries as there is a high demand for these materials in hybrid electric vehicles and consumer electronics^1–6^. These materials have to be relatively low cost, large density of Li^+^ ions and safety requirements. Considerable research activity has been devoted to identify alternative promising cathode materials such as Li_2_MSiO_4_ (M = Fe, Mn and Co)^7–14^, Li_3_V(MoO_4_)_3_^15^, LiFeSO_4_F^16^, Li_2_FeP_2_O_7_^17^ and Li_7_Mn(BO_3_)_3_^18^. Nevertheless, the current materials has many challenges such as poor electrochemical performance and low electrical conductivity to satisfy the practical applications. Thus, the search for novel materials is needed to overcome those challenges and satisfy the growing energy demand.

“Li-rich” Li_3_NbO_4_ has been studied as the host material for a new class of high-capacity positive cathode materials for rechargeable lithium batteries^19–21^. Recent experimental investigations show that manganese-substituted Li_3_NbO_4_ can provide a large reversible capacity of approximately 300 mAg^−119^. Naoaki *et al*.^20^ studied experimentally the substitution of V^3+^ ions in Li_3_NbO_4_ and observed a reversible capacity of approximately 230 mAhg^−1^. A significance increase in Li^+^ ion conductivity has been observed in Li_3_NbO_3_ with increasing concentration of Ni^2+^ ions sharing Li^+^ and Nb^5+^ sites^21^. Though these studies report the capacity of substituted Li_3_NbO_4_, there are no experimental or theoretical studies available for pristine Li_3_NbO_4_.

Static atomistic simulation methods based on the classical pairwise potentials can provide useful information on defect properties including cation mixing and lithium ion migration paths together with activation energies. This computational methodology has been successfully applied to a range of battery materials including LiFePO_4_^22^, Li_2_FeSiO_4_^9^ and Li_2_MnSiO_4_^12^. We have recently employed this methodology to examine the defects, lithium ion diffusion and the solution of a variety of dopants on the Li_5_FeO_4_^23^, Li_2_CuO_2_^24^, Li_9_V_3_(P_2_O_7_)_3_(PO_4_)^25^, Li_2_SnO_3_^26^ and Li_2_TiO_3_^27^ battery materials. In this study, atomistic simulation techniques as implemented in the GULP code^28^ are employed to calculate the formation energies for the intrinsic defects, the lithium ion conduction pathways and the solution of tetravalent dopants for introducing additional lithium in Li_3_NbO_4_. Density functional theory (DFT) simulations as implemented in the CASTEP code^29^ were used to study the electronic properties of doped-and undoped-Li_3_NbO_4_.

## Results and Discussion

### Structural Modelling

**Figure 1:**
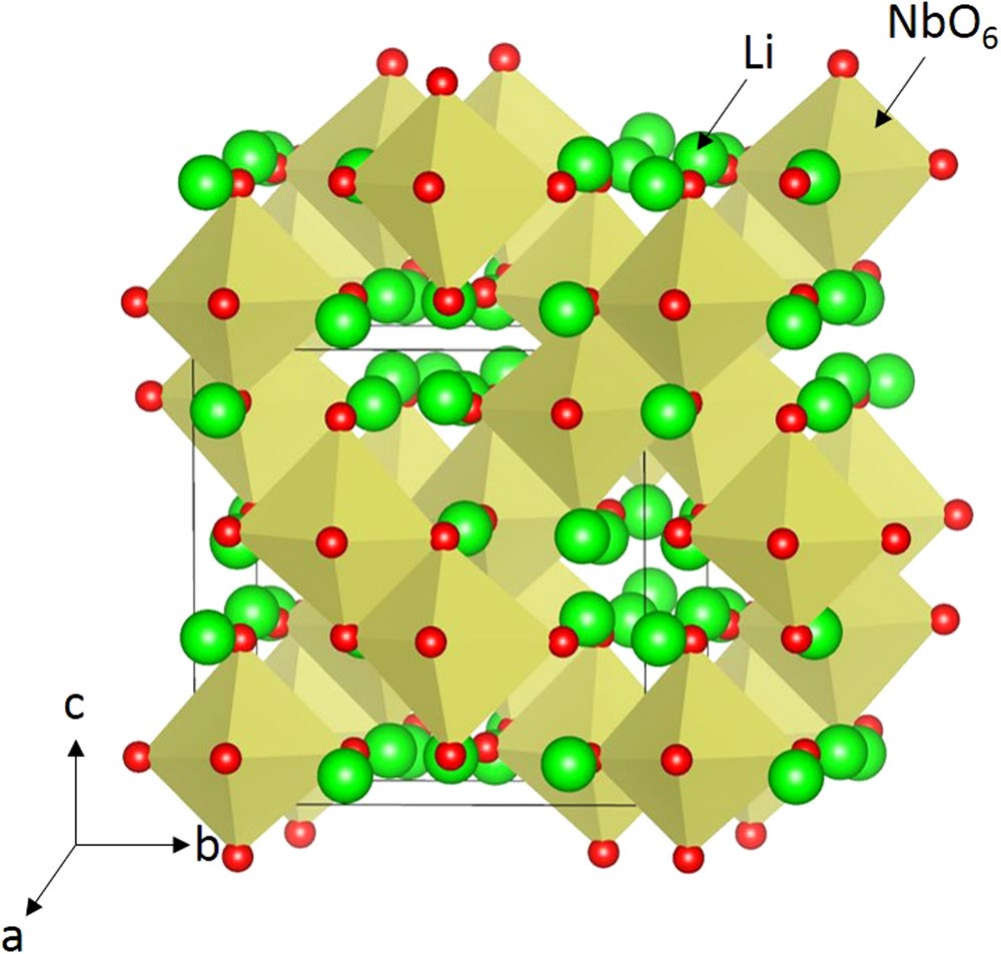
Crystal structure of Li_3_NbO_4_ (space group ${\rm{I}}\bar{4}3{\rm{m}}$).

Crystallographic structure of Li_3_NbO_4_ is cubic with space group ${\rm{I}}\bar{4}3{\rm{m}}$ (lattice parameters a = b = c = 8.415 Å, α = β = γ = 90°) as determined by Ukei *et al*.^30^. Figure 1 exhibits this crystal structure and the bonding nature of cations with Nb forming edge-shared NbO_6_ octahedra in the lattice. First, we reproduced the experimental crystal structure of Li_3_NbO_4_ to test the quality of the classical pairwise potential parameters used in this study (refer to Table S1 in the supplementary information). An excellent agreement was obtained between the calculated and experimental lattice parameters (refer to Table 1).

**Table 1:** Calculated and Experimental Structural Parameters for cubic (${\rm{I}}\bar{4}3{\rm{m}}$) Li_3_NbO_4._

Parameter	Calc	Expt^30^	|∆|(%)
a = b = c (Å)	8.412	8.415	0.03
α = β = γ (°)	90.0	90.0	0.00

### Intrinsic defects

Next we calculated the vacancy and interstitial defect formation energies to calculate the Frenkel and Schottky-type defect formation energies in Li_3_NbO_4_. These defects are useful to examine the electrochemical properties of Li_3_NbO_4_. The following reaction equations written using Kröger-Vink notation^31^ represent the Frenkel, Schottky and anti-site type defects.

1 $${\rm{Li}}\,\mathrm{Frenkel}:\,{{\rm{Li}}}_{{\rm{Li}}}^{{\rm{X}}}\to {V}_{{\rm{Li}}}^{{\prime} }+{{\rm{Li}}}_{{\rm{i}}}^{\bullet }$$

2 $${\rm{O}}\,\mathrm{Frenkel}:\,{{\rm{O}}}_{{\rm{O}}}^{{\rm{X}}}\to {V}_{{\rm{o}}}^{\bullet \bullet }+{{\rm{O}}}_{{\rm{i}}}{\prime\prime}$$

3 $${\rm{Nb}}\,\mathrm{Frenkel}:\,{V}_{{\rm{Nb}}}^{{\rm{X}}}\to {V}_{{\rm{N}}{\rm{b}}}{\prime\prime\prime\prime\prime }+{{\rm{Nb}}}_{{\rm{i}}}^{\bullet \bullet \bullet \bullet \bullet }$$

4 $$\mathrm{Schottky}:\,3\,{{\rm{Li}}}_{\mathrm{Li}\,}^{{\rm{X}}}+{{\rm{Nb}}}_{{\rm{Nb}}}^{{\rm{X}}\,}+4\,{{\rm{O}}}_{{\rm{O}}}^{{\rm{X}}}\to 3\,{V}_{{\rm{L}}{\rm{i}}}{\prime }+{V}_{{\rm{N}}{\rm{b}}}{\prime\prime\prime\prime\prime}+4\,{V}_{{\rm{O}}}^{\bullet \bullet }+{{\rm{Li}}}_{3}{{\rm{NbO}}}_{4}$$

5 $${{\rm{Li}}}_{2}{\rm{O}}\,\mathrm{Schottky}:\,2\,{{\rm{Li}}}_{{\rm{Li}}}^{{\rm{X}}}+{{\rm{O}}}_{{\rm{O}}}^{{\rm{X}}\,}\to 2\,{V}_{{\rm{L}}{\rm{i}}}{\prime }+{V}_{{\rm{O}}}^{\bullet \bullet }+{{\rm{Li}}}_{2}{\rm{O}}$$

6 $${\rm{Li}}/{\rm{Nb}}\,{\rm{antisite}}\,({\rm{isolated}}):{{\rm{Li}}}_{{\rm{Li}}}^{{\rm{X}}}+{V}_{{\rm{Nb}}}^{{\rm{X}}\,}\to {{\rm{Li}}}_{{\rm{N}}{\rm{b}}}{{\prime\prime\prime\prime} }+{{\rm{Nb}}}_{{\rm{Li}}}^{\bullet \bullet \bullet \bullet }$$

7 $${\rm{Li}}/{\rm{Nb}}\,{\rm{antisite}}\,({\rm{cluster}}):{{\rm{Li}}}_{{\rm{Li}}}^{{\rm{X}}}+{{\rm{Nb}}}_{{\rm{Nb}}}^{{\rm{X}}}\to {\{{{\rm{Li}}}_{{\rm{N}}{\rm{b}}}{\prime\prime\prime\prime}:{{\rm{Nb}}}_{{\rm{Li}}}^{\bullet \bullet \bullet \bullet }\}}^{{\rm{X}}}$$

**Figure 2:**
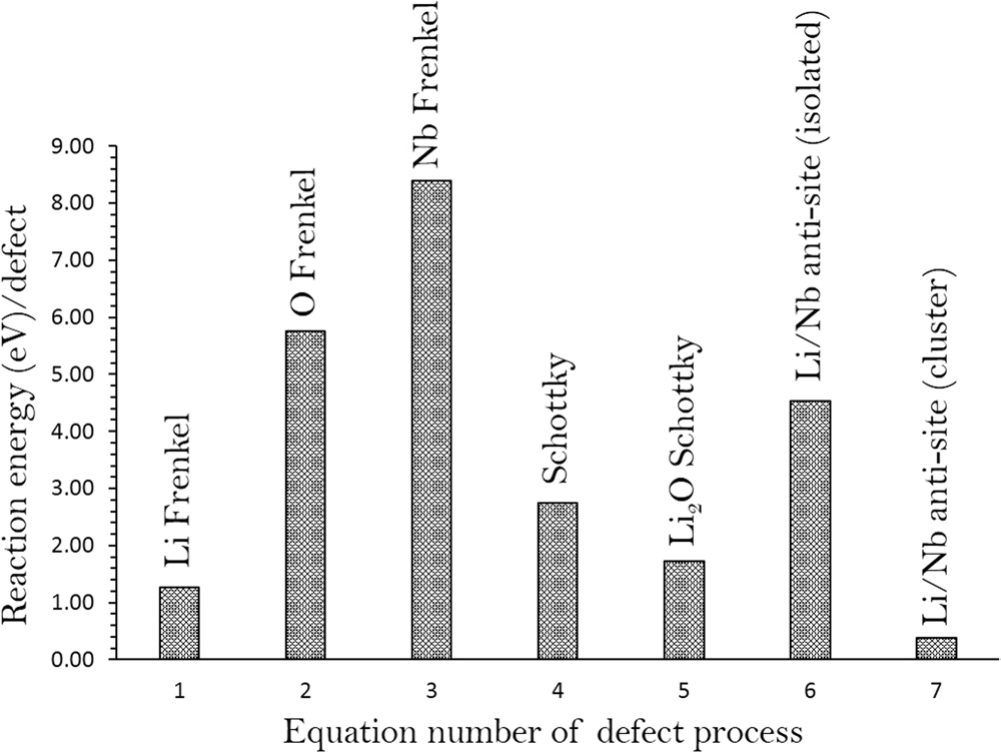
Energetics of intrinsic defect process in cubic Li_3_NbO_4_.

Figure 2 shows energies calculated for these intrinsic defect equations. The most favourable intrinsic defect is the Li-Nb anti-site (eq. 7). This result suggests that a small percentage of Li on Nb sites (${{\rm{Li}}}_{{\rm{N}}{\rm{b}}}{{\prime\prime\prime\prime} }$) and Nb on Li sites (${{\rm{Nb}}}_{{\rm{Li}}}^{\bullet \bullet \bullet \bullet })$ would be observed. The precise concentration depends on the temperature and synthetic procedure. Anti-site defect was noted in a variety of other Li battery materials particularly during cycling^9,12,32–35^.

The Li Frenkel is the second most favourable intrinsic defect. The Li_2_O Schottky (relation 5) is calculated be 1.73 eV per defect (see Table S2). This defect process leads to further ${V}_{Li}{\prime}$ and ${V}_{O}^{\bullet \bullet }$ however at high temperatures. The other defect processes exhibit highly endoergic suggesting that they are unlikely to form.

### Lithium ion-diffusion

**Figure 3:**
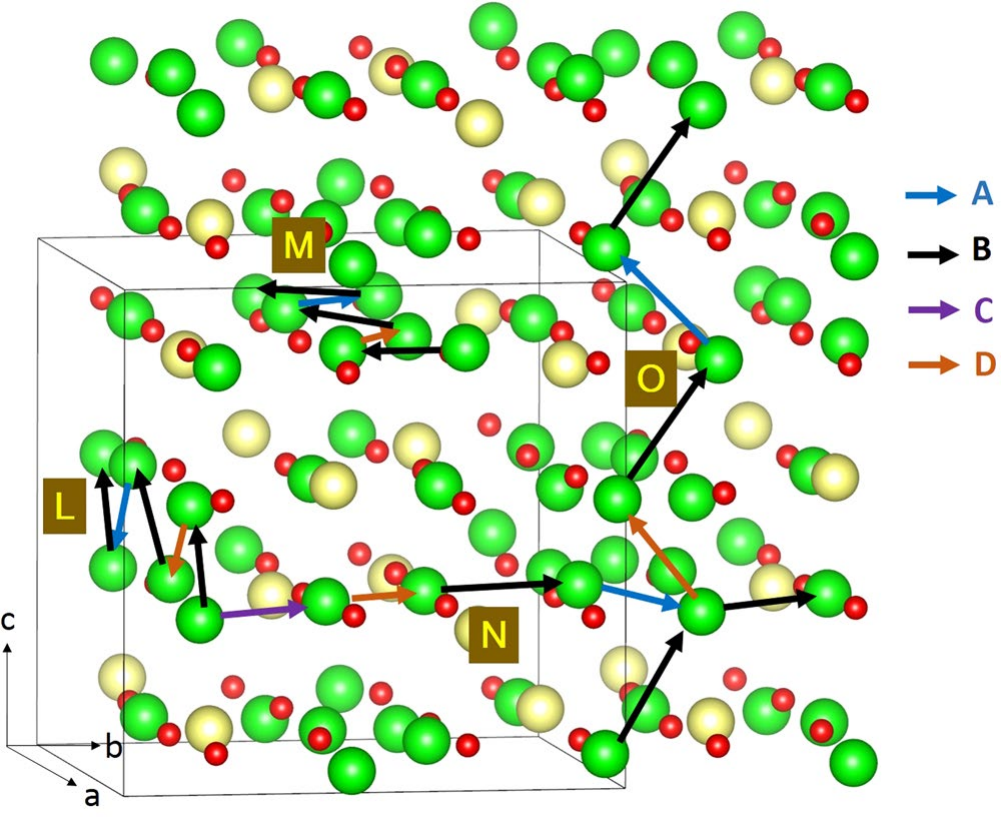
Possible long range lithium vacancy migration paths considered. Green, yellow and red colours correspond to Li, Nb, and O atoms respectively. Long range paths are labelled as L, M, N and O.

**Figure 4:**
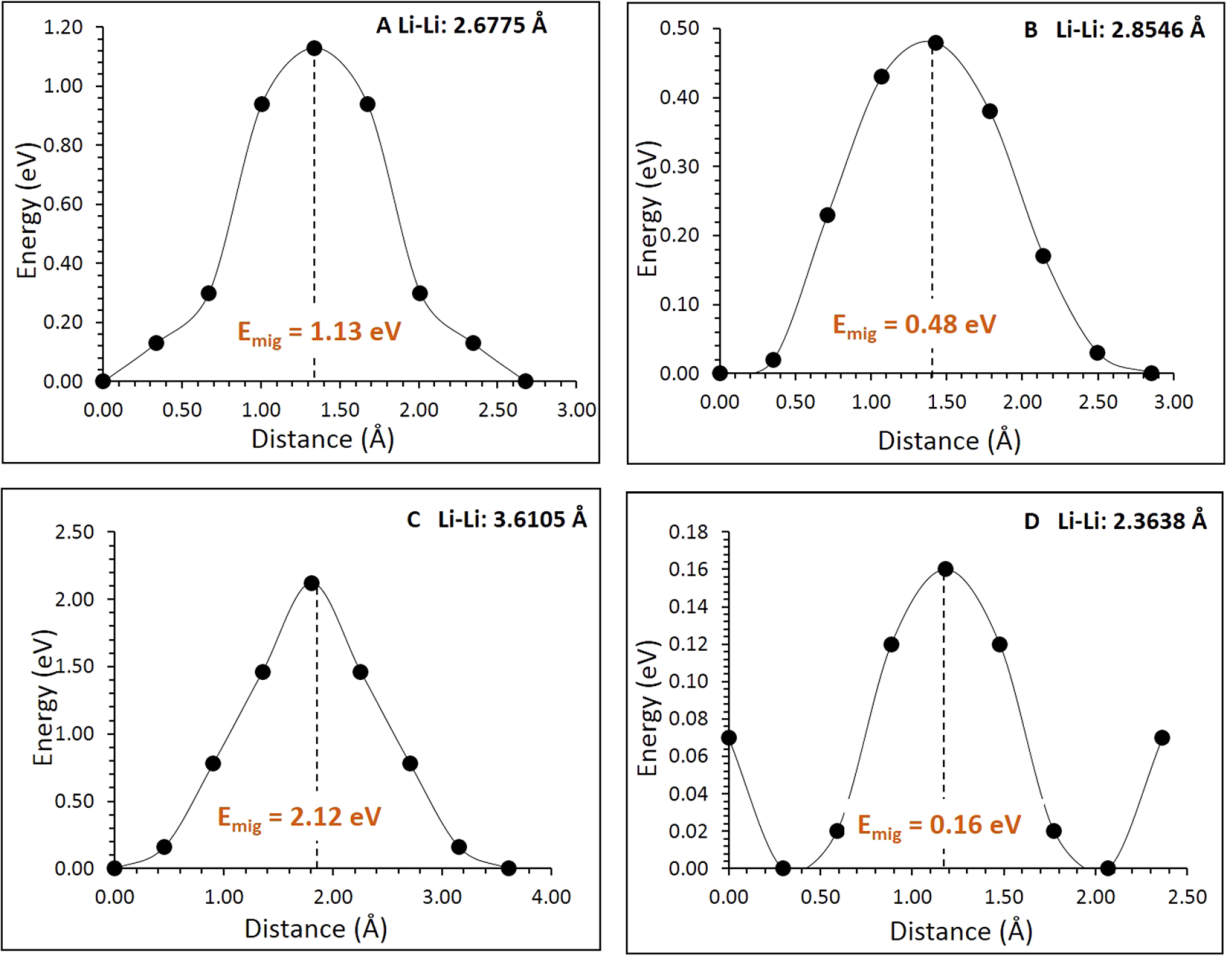
Four different energy profiles [as shown in Fig. 3] of Li vacancy hopping between two adjacent Li sites in Li_3_NbO_4_.

An essential requirement for a potential high-capacity cathode material in lithium ion batteries is the lower activation energy for lithium ion migration. In general, it is difficult to determine the paths of lithium ion diffusion and their activation energies experimentally. However, using static atomistic simulation, it is possible to construct possible long range Li ion diffusion paths. For the Li vacancy migration, four distinct local Li hops (refer to Fig. 3) were calculated. Activation energies together with the Li-Li separation are reported in Table 2 and corresponding energy profile diagrams are shown in Fig. 4. Long range Li ion diffusion paths connecting local Li hops were constructed. Table 3 summarizes the possible long range paths together with the corresponding overall activation energies. Additional long range paths were considered but their overall activation energies were not less than the values reported in the Table 3. We have identified three zig-zag long range paths (along *ab*, *bc* and *ac* planes) with the lower overall activation energy of 1.13 eV. In all four long range paths, there are two local Li-Li hops with lower activation energies of 0.16 eV and 0.48 eV. However, higher activation energy local Li-Li hops increase overall activation energies. There are no theoretical calculations available in the literature providing details on the diffusion mechanism and the migration energies. It is thus expected that this investigation will stimulate experimentalists to look at this material as a viable cathode material.

**Table 2:** Calculated Li-Li separations and activation energies for the lithium ion migration between two adjacent Li sites refer to Fig. 3.

Migration path	Li-Li separation (Å)	Activation energy (eV)
A	2.6775	1.13
B	2.8546	0.48
C	3.6105	2.12
D	2.3638	0.16

**Table 3:** Possible long range Li ion diffusion paths and their corresponding overall activation energies.

Long range path	Direction	Overall activation energy (eV)
L: B → D → B → A → B	along *ac* plane	1.13
M: B → D → B → A → B	along *ab* plane	1.13
N: C → D → B → A → B	along *ac* plane	2.12
O: B → D → B → A → B	along *bc* plane	1.13

### Tetravalent doping

The performance of a promising new cathode material depends on its high energy density and power density with appropriate safety. Incorporation of extra lithium will improve the capacity of the as-prepared material and increase the use of Li_3_NbO_4_ as a potential cathode material in the lithium batteries. An efficient defect engineering strategy to increase the concentration of lithium as Li interstitials is by doping tetravalent cations on Nb site. Similar computational strategy was used in Li_2_MnSiO_4_ cathode material in which Al and Ga were doped on Si site^12^. Here we considered the solution of ${{\rm{RO}}}_{2}$ (*R* = Si, Ge, Ti, Zr and Ce) using the following process (in Kröger-Vink notation):

8 $$2\,{{\rm{RO}}}_{2}+2{{\rm{Nb}}}_{{\rm{Nb}}}^{{\rm{X}}}+{{\rm{Li}}}_{2}{\rm{O}}\to 2\,{{\rm{R}}\prime }_{{\rm{Nb}}}+2\,{{\rm{Li}}}_{{\rm{i}}}^{\bullet }+{{\rm{Nb}}}_{2}{{\rm{O}}}_{5}$$

**Figure 5:**
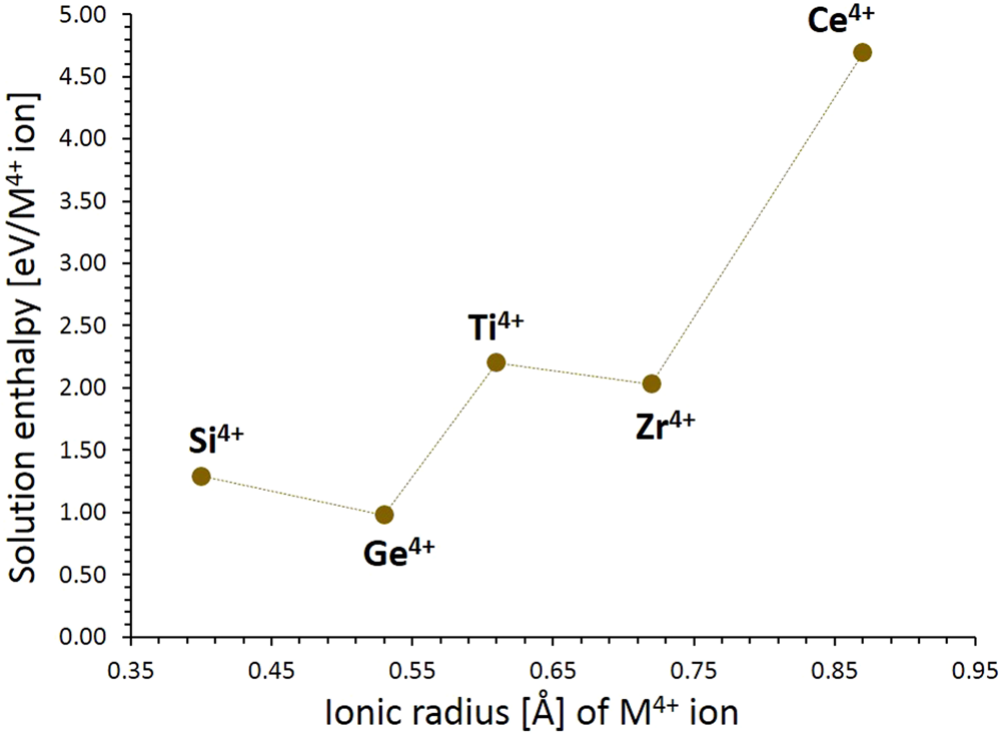
Enthalpy of solution of *R*O_2_ (*R* = Si, Ge, Ti, Zr, and Ce) with respect to the R^4+^ ionic radius in Li_3_NbO_4_.

Solution energies of ${{\rm{RO}}}_{2}$ is reported in Fig. 5. The most favourable dopant solution energy (0.98 eV) is calculated for GeO_2_. This indicates that a possible way of introducing extra lithium into Li_3_NbO_4_ is by doping Ge^4+^ on Nb site, although the precise concentration of Ge incorporation is unpredictable. The possible composition of Ge-doped Li_3_NbO_4_ would be Li_3+x_Nb_1-x_Ge_x_O_4_ (x = 0.0–1.0). The solution energy for SiO_2_ is 1.29 eV, suggesting that Si is also a candidate dopant. Solution energies for TiO_2_ and ZrO_2_ are 2.20 eV and 2.03 eV respectively. Highly endothermic (4.69 eV) solution energy is calculated for CeO_2_.

**Figure 6:**
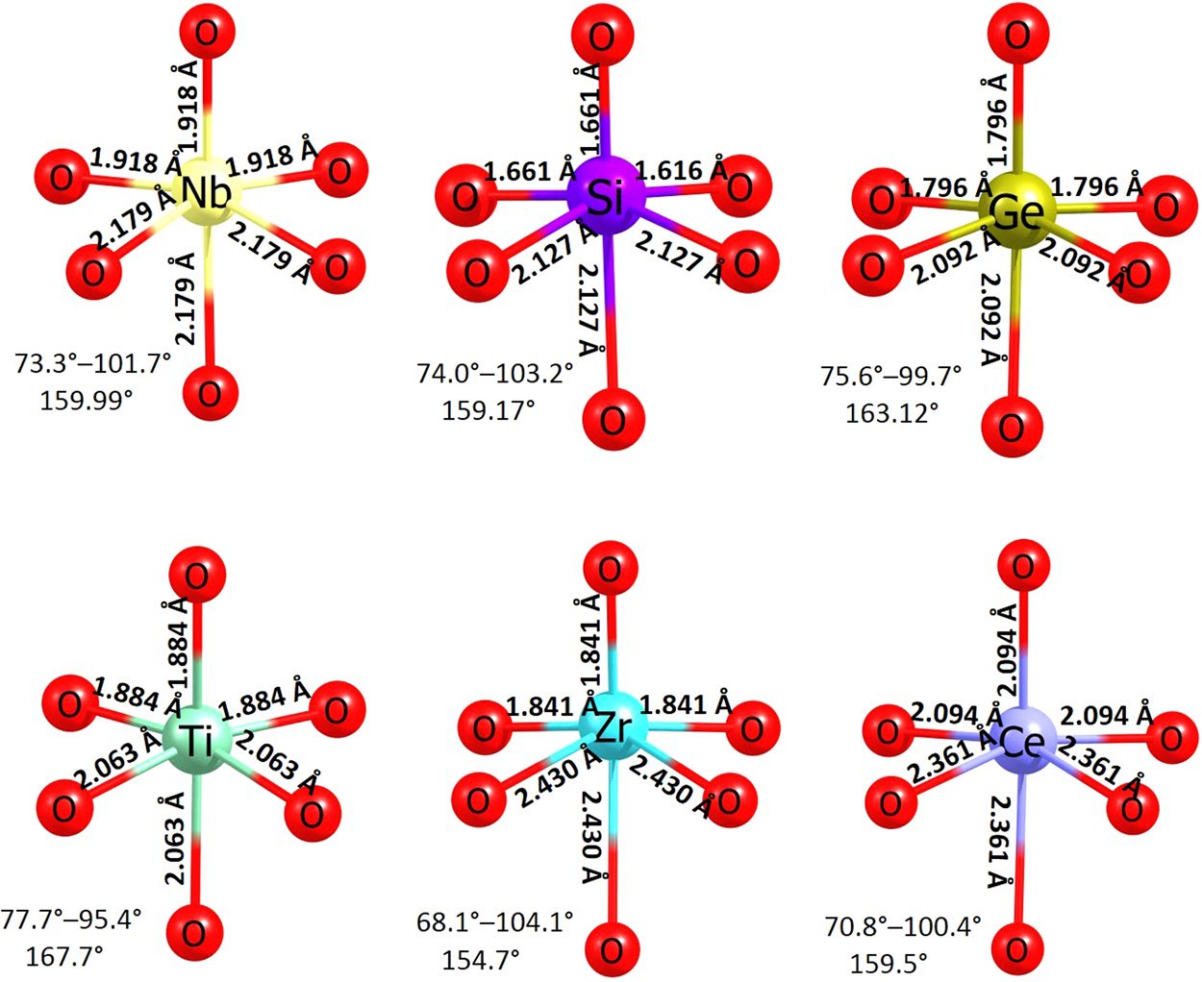
Octahedral NbO_6_ unit in the relaxed structure of undoped Li_3_NbO_4_ and the coordination formed by the dopants on the Nb site with oxygen neighbour.

Local coordination of Nb with oxygen, Nb-O bond lengths and O-Nb-O bond angles in the relaxed configuration of undoped Li_3_NbO_4_ and the dopants occupying Nb site, is reported in Fig. 6. The ionic radius of Nb^5+^ is 0.64 Å in octahedral environment. The ionic radius of Ge^4+^ is smaller by 0.11 Å than that of Nb^5+^. In the GeO_6_ unit, all six Ge-O bonds are shorter than the Nb-O bonds present in the pristine Li_3_NbO_4._ This is because of the smaller cation size of Gi^4+^ which strongly perturbs the oxygen ions leading to stronger bonds with O atoms. The second most favourable solution energy is calculated for Si^4+^. Its ionic radius (0.40 Å) is 0.24 Å, shorter than that of Nb^5+^. This is reflected in the shorter bond distances. However, due to its somewhat larger cation mismatch, the solution energy increases slightly. In the TiO_6_ unit, all six Ti-O bond lengths are shorter than those observed in the NbO_6_ unit. Notably, bond angles deviate significantly reflecting in the solution energy. In the ZrO_6_ unit, three shorter and three longer Zr-O bonds are observed. Nevertheless, bond angles are shorter than the other octahedral units. Solution energy is slightly lower than that calculated for Ti. The difference in the solution energies is also dependent on the electronic structure of the dopants. There is a significant rise in the solution energy (4.69 eV) for CeO_2_ due to its larger ionic radius reflecting in the longest bond lengths and shortest bond angles.

### Densities of states

**Figure 7:**
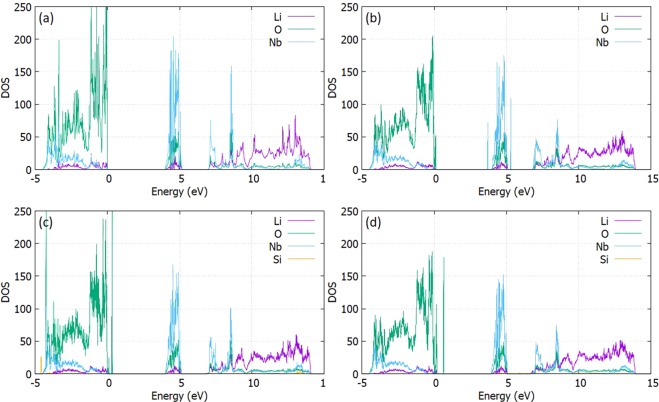
The Li_3_NbO_4_ PDOS for the (**a**) Perfect cell (**b**) The Li^+^ Interstitial defect (**c**) The Si^4+^-doped cell (**d**) The Si^4+^-doped with a Li^+^ interstitial defect.

The electronic structure of Li_3_NbO_4_ is investigated using DFT. Figure 7(a) presents the electronic structure with the O^2−^ p-states set to the Fermi level forming a band gap of 6.95 eV where the Nb^5+^ d-states dominate the conduction band (Refer to SI Fig. 1 for the exact contribution of orbitals). In addition, a contribution of in-gap states is located at 4.0 eV from the valence band with a width of 1.15 eV, mainly attributed at the O^2−^ p-states and the Nb^5+^ d-states as well. Furthermore, Li^+^ is calculated to have the strongest density in the conduction band. Overall, it is expected that the electronic conduction of Li_3_NbO_4_ not to be the main conduction mechanism. Introducing Li^+^ interstitials in the crystal does not significantly affect the profile of the total DOS, however, a small tail of the valence band is observed in conjunction with additional Nb^5+^ contribution close to the mid-gap state region. Doping with tetravalent elements will increase the formation of Li^+^ ion hosted in interstitial sites of the crystal. The increased Li^+^ concentration in the crystal does not promote the increase of the total Li^+^ contribution. The Si^4+^-doped Li_3_NbO_4_ corresponds to the lowest solution enthalpy while it is characterized by the smallest ionic radius of all the dopants examined. Figure 1(c,d) shows the DOS for the Si^4+^ doped supercell before and after the introduction of one Li^+^ interstitial respectively. We expect the formation of new states due to the dopant reaction with the host lattice. This should be considered as both a structural distortion as well as a point of association with the electronic configuration between neighbouring atoms. The doping effect relates to additional contributions close to the conduction band as well as in the middle of the gap as well. The defect pairs of ${{\rm{G}}{\rm{e}}}_{{\rm{N}}{\rm{b}}}{\prime }$ and ${\{{{\rm{G}}{\rm{e}}}_{{\rm{N}}{\rm{b}}}{\prime }:{\rm{L}}{{\rm{i}}}_{{\rm{i}}}^{\bullet }\}}^{{\rm{X}}}$, ${{\rm{T}}{\rm{i}}}_{{\rm{N}}{\rm{b}}}{\prime }$ and ${\{{{\rm{T}}{\rm{i}}}_{{\rm{N}}{\rm{b}}}{\prime }:{\rm{L}}{{\rm{i}}}_{{\rm{i}}}^{\bullet }\}}^{{\rm{X}}}$, ${{\rm{Z}}{\rm{r}}}_{{\rm{N}}{\rm{b}}}{\prime }$ and ${\{{{\rm{Z}}{\rm{r}}}_{{\rm{N}}{\rm{b}}}{\prime }:{\rm{L}}{{\rm{i}}}_{{\rm{i}}}^{\bullet }\}}^{{\rm{X}}}$ and ${Ce}_{Nb}{\prime }$ and ${\{{{\rm{C}}{\rm{e}}}_{{\rm{N}}{\rm{b}}}{\prime }:{\rm{L}}{{\rm{i}}}_{{\rm{i}}}^{\bullet }\}}^{{\rm{X}}}$ have been also examined. Unsurprisingly, minimum and maximum intensity corresponds to those of the smallest and biggest radius respectively (Refer to Fig. 2, of the SI for the dopants considered). Additionally, the interstitial mechanism tends to increase the dopant contribution whereas a slight non – uniformity of the valence band near the Fermi level with extra O^2−^ states in the gap is observed. As the dominant diffusion mechanism corresponds to the Li^+^ Frenkel, we have also tested the doped structure with a Li^+^ vacancy (Refer to Fig. 3, SI). The defect pairs are ${{\rm{G}}{\rm{e}}}_{{\rm{N}}{\rm{b}}}{\prime }$ and $\{{{\rm{G}}{\rm{e}}}_{{\rm{N}}{\rm{b}}}{\prime }:{\rm{L}}{{\rm{i}}}_{{\rm{i}}}^{\bullet }\},$ ${{\rm{T}}{\rm{i}}}_{{\rm{N}}{\rm{b}}}{\prime }$ and $\{{{\rm{T}}{\rm{i}}}_{{\rm{N}}{\rm{b}}}{\prime }:{\rm{L}}{{\rm{i}}}_{{\rm{i}}}^{\bullet }\}$, ${{\rm{Z}}{\rm{r}}}_{{\rm{N}}{\rm{b}}}{\prime }$ and $\{{{\rm{Z}}{\rm{r}}}_{{\rm{N}}{\rm{b}}}{\prime }:{\rm{L}}{{\rm{i}}}_{{\rm{i}}}^{\bullet }\}$ and ${{\rm{C}}{\rm{e}}}_{{\rm{N}}{\rm{b}}}{\prime }$ and $\{{{\rm{C}}{\rm{e}}}_{{\rm{N}}{\rm{b}}}{\prime }:{\rm{L}}{{\rm{i}}}_{{\rm{i}}}^{\bullet }\}$. Overall, the materials behaviour remains similar to the interstitial presence and we expect the electronic structure with respect to the total Frenkel mechanism to remain unaffected, with the additional contributions to be attributed to the dopants.

### Summary

In this study, atomistic simulation technique was applied to examine the defect energetics, lithium ion diffusion and doping behaviour as they are relevant when assessing Li_3_NbO_4_ as a promising lithium battery cathode material. The lowest energy defect process is Li-Nb anti-site defect indicating that a small percentage of Nb on Li sites would be observed at operating temperatures. Two dimensional long range Li ion diffusion path was calculated with the lowest overall migration energy of 1.13 eV, suggesting slightly lower Li mobility at low temperatures, but higher diffusion would be observed at operating temperatures. As compared to recent studies the migration energy barrier is higher^23–27,36^. An advantage of Li_3_NbO_4_ is the directional diffusion mechanism, which is not the case for Li_5_FeO_4_ and Na_2_MnSiO_4_ considered previously^23,36^. An important feature of Li_3_NbO_4_ is its ease to incorporate dopants with the solution energies of ${{\rm{RO}}}_{2}$ (*R* = Si, Ge, Ti, Zr and Ce) calculated to create extra lithium in this material. The energetically favourable (i.e. lowest solution energy of RO_2_) being Ce. These interesting results presented here demonstrate that experimental work should be encouraged on this important lithium ion battery material. Additionally, mixed computational techniques can be employed to calculate the optimum doping conditions and the impact on self-diffusion^37^.

### Methods

Defect and Li ion migration calculations were performed using the classical pair wise potential method. The GULP code^28^, which is based on the classical Born model description of an ionic crystal lattice was used. In this method, two types of ionic interactions were considered. The long-range attractions are based on the Coulombic forces. The short-range repulsive forces consisting of electron-electron repulsion and van der Waals interactions were modelled using Buckingham potentials (refer to Table S1). Geometry optimisation (both positions of atoms and lattice constants) was performed using the Broyden-Fletcher-Goldfarb-Shanno (BFGS) algorithm^38^. Lattice relaxation around the point defects and the migrating Li^+^ ions was modelled using the Mott-Littleton method^39^. To calculate the Li ion diffusion we considered two adjacent vacancy sites as initial and final configurations. Here, the activation energy of Li ion diffusion is defined as the local maximum energy along this diffusion path. In the current methodology, the defect enthalpies will be overestimated due to the full charge ionic model with dilute limit used in the present study, but the trends in the defect energies will be consistent.

Electronic properties of doped- and undoped-Li_3_NbO_4_ were calculated using a plane wave DFT code CASTEP^29,40^. All supercells were optimized to the most stable configuration with the plane wave basis set to a cut-off of 500 eV. All the Li_3_NbO_4_ supercells were simulated using a 2 × 2 × 2 Monkhorst-Pack (MP)^41^ k-point grid within a supercell containing 128 atoms. The non–defective, defective and doped structures have been modelled under constant pressure conditions. We apply the exchange and correlation interactions in the crystallographic structure of the material using the formulation with the corrected density functional of Perdew, Burke and Ernzerhof (PBE)^42^ within the generalized gradient approximation (GGA) in conjunction with ultrasoft pseudopotentials^43^. For the electronic structure calculations and output visualization, the OPTADOS^44,45^ tool has been employed.

## Supplementary information

Defects, Lithium Mobility and Tetravalent Dopants in the Li3NbO4 Cathode Material
