# Supplementary material for: Defects, Lithium Mobility and Tetravalent Dopants in the Li3NbO4 Cathode Material
Source: Sci Rep. 2019 Feb 18;9:2192. doi: 10.1038/s41598-018-37466-x (PMC6379435; doi:10.1038/s41598-018-37466-x)
Supplement: Supplementary file 1 — Defects, Lithium Mobility and Tetravalent Dopants in the Li3NbO4 Cathode Material [file 41598_2018_37466_MOESM1_ESM.docx]

**Supporting Information**

[**Defects, Lithium Mobility and Tetravalent Dopants in the Li_3_NbO_4_ Cathode Material**](http://ma.ecsdl.org/content/MA2010-03/1/440.short)

Navaratnarajah Kuganathan,^1,a^ Apostolos Kordatos,^2^ Nikolaos Kelaidis,^2^ and Alexander Chroneos^1,2,b)^

^1^Department of Materials, Imperial College London, London, SW7 2AZ, United Kingdom

^2^Faculty of Engineering, Environment and Computing, Coventry University, Priory Street, Coventry CV1 5FB, United Kingdom

Corresponding authors, e-mails: a) n.kuganathan@imperial.ac.uk

b) [alexander.chroneos@imperial.ac.uk](mailto:alexander.chroneos@imperial.ac.uk)

**Table S1**. Interatomic potential parameters used in the atomistic simulations of Li_3_NbO_4_.

Two-body [Φ*_ij_* (*r_ij_*) = *A_ij_* exp (− *r_ij_* /*ρ_ij_*) − *C_ij_ / r_ij_*^6^]

| Interaction | *A* (eV) | *ρ* (Å) | *C* (eV·Å^6^) | Y (e) | K (eV·Å^-2^) |
| --- | --- | --- | --- | --- | --- |
| Li^+^–O^2−^ | 950.000 | 0.2610 | 0.00 | 1.000 | 99999 |
| Nb^5+^–O^2−^ | 1425.00 | 0.3650 | 0.49 | 5.000 | 99999 |
| O^2−^–O^2−^ | 22764.30 | 0.1490 | 27.88 | –2.900 | 70.00 |
| Si^4+^–O^2–^ | 1283.91 | 0.32052 | 10.66 | 4.000 | 99999 |
| Ge^4+^–O^2–^ | 1497.3996 | 0.325646 | 16.00 | 4.000 | 99999 |
| Ti^4+^–O^2–^ | 5111.70 | 0.2625 | 0.00 | –0.100 | 314.92 |
| Zr^4+^–O^2–^ | 985.869 | 0.3760 | 0.00 | 1.350 | 169.617 |
| Ce^4+^–O^2–^ | 1986.83 | 0.3511 | 20.40 | 7.700 | 291.75 |

**Table S2.** Energetics of intrinsic defect process in Li_3_NbO_4_

| Defect process/equation | Reaction energy/eV | Reaction energy per defect/eV |
| --- | --- | --- |
| Li Frenkel /1 | 2.54 | 1.27 |
| O Frenkel /2 | 5.76 | 2.88 |
| Nb Frenkel /3 | 16.78 | 8.39 |
| Schottky /4 | 21.88 | 2.74 |
| Li_2_O Schottky/5 | 5.18 | 1.73 |
| Li/Nb antisite (isolated) /6 | 9.06 | 4.53 |
| Li/Nb antisite (cluster) /7 | 0.78 | 0.39 |


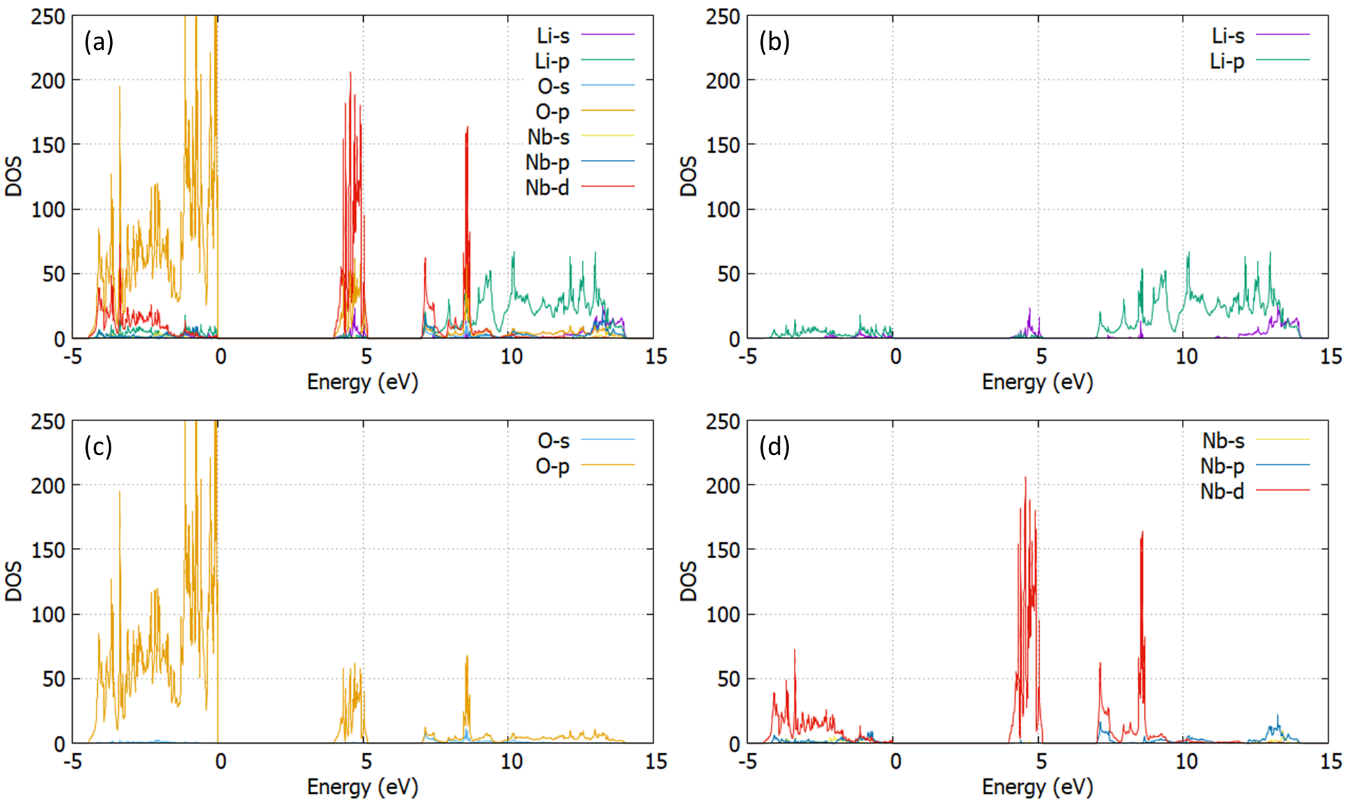


**Figure S1.** The PDOS for the non-defective Li_3_NbO_4_ structure with respect to (a) The complete contribution of orbitals (b) The Li^+^ orbitals only (c) The O^2+^ orbitals only (d) The Nb^5+^ orbitals only


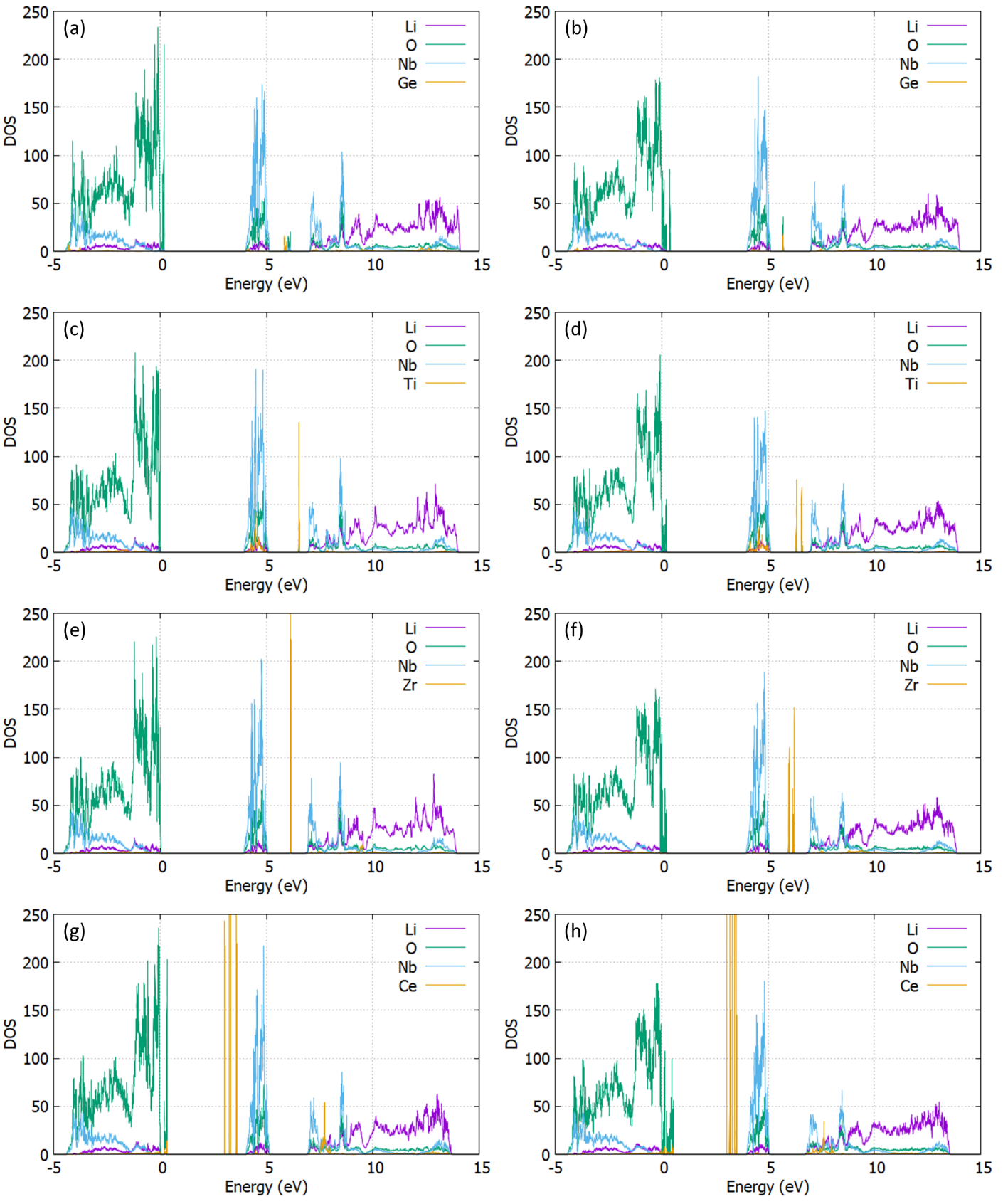


**Figure S2.** The PDOS for the doped supercell with respect to (a) Ge^4+^ doping (b) Ge^4+^ doping with one Li^+^ interstitial (c) Ti^4+^ doping (d) Ti^4+^ doping with one Li^+^ interstitial (e) Zr^4+^ doping (e) Zr^4+^ doping with one Li^+^ interstitial (g) Ce^4+^ doping (h) Ce^4+^ doping with one Li^+^ interstitial


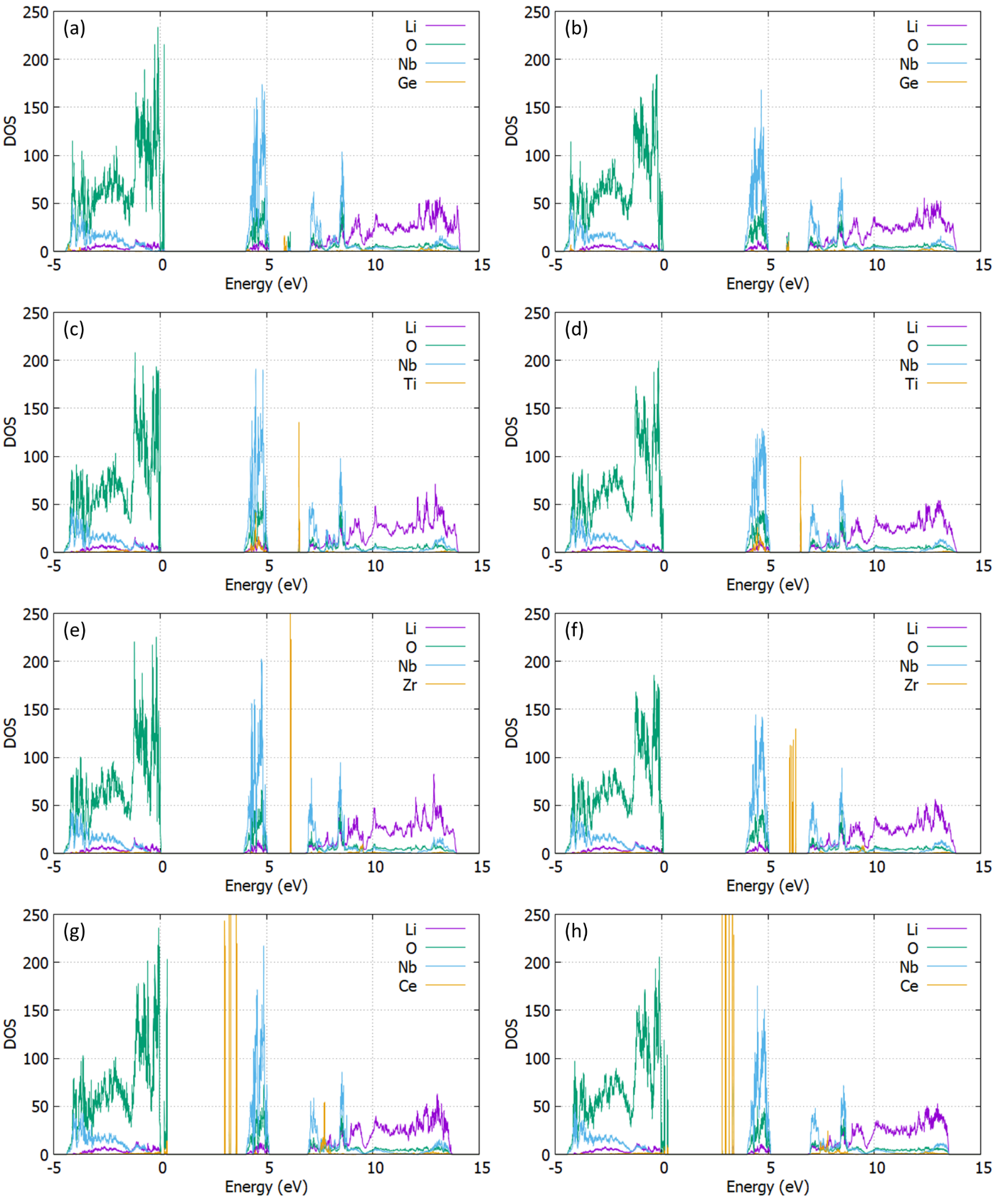


**Figure S3** The PDOS for the doped supercell with respect to (a) Ge^4+^ doping (b) Ge^4+^ doping with one Li^+^ vacancy (c) Ti^4+^ doping (d) Ti^4+^ doping with one Li^+^ vacancy (e) Zr^4+^ doping (e) Zr^4+^ doping with one Li^+^ vacancy (g) Ce^4+^ doping (h) Ce^4+^ doping with one Li^+^ vacancy
